# CD38 deficiency suppresses adipogenesis and lipogenesis in adipose tissues through activating Sirt1/PPARγ signaling pathway

**DOI:** 10.1111/jcmm.13297

**Published:** 2017-08-16

**Authors:** Ling‐Fang Wang, Lian‐Jie Miao, Xiao‐Nv Wang, Cong‐Cong Huang, Yi‐Song Qian, Xuan Huang, Xiao‐Lei Wang, Wan‐Zhu Jin, Guang‐Ju Ji, Mingui Fu, Ke‐Yu Deng, Hong‐Bo Xin

**Affiliations:** ^1^ Institute of Translational Medicine Nanchang University Nanchang China; ^2^ School of Life Sciences Nanchang University Nanchang China; ^3^ Institute of Zoology Chinese Academy of Sciences Beijing China; ^4^ National Laboratory of Biomacromolecules Institute of Biophysics Chinese Academy of Sciences Beijing China; ^5^ Department of Basic Medical Science Shock/Trauma Research Center School of Medicine University of Missouri Kansas City Kansas City MO USA

**Keywords:** CD38, Sirt1, peroxisome proliferator‐activated receptor γ, adipogenesis, lipogenesis

## Abstract

It has been recently reported that CD38 was highly expressed in adipose tissues from obese people and CD38‐deficient mice were resistant to high‐fat diet (HFD)‐induced obesity. However, the role of CD38 in the regulation of adipogenesis and lipogenesis is unknown. In this study, to explore the roles of CD38 in adipogenesis and lipogenesis *in vivo* and *in vitro*, obesity models were generated with male CD38^−/−^ and WT mice fed with HFD. The adipocyte differentiations were induced with MEFs from WT and CD38^−/−^ mice, 3T3‐L1 and C3H10T1/2 cells *in vitro*. The lipid accumulations and the alternations of CD38 and the genes involved in adipogenesis and lipogenesis were determined with the adipose tissues from the HFD‐fed mice or the MEFs, 3T3‐L1 and C3H10T1/2 cells during induction of adipocyte differentiation. The results showed that CD38^−/−^ male mice were significantly resistant to HFD‐induced obesity. CD38 expressions in adipocytes were significantly increased in WT mice fed with HFD, and the similar results were obtained from WT MEFs, 3T3‐L1 and C3H10T1/2 during induction of adipocyte differentiation. The expressions of PPARγ, AP2 and C/EBPα were markedly attenuated in adipocytes from HFD‐fed CD38^−/−^ mice and CD38^−/−^ MEFs at late stage of adipocyte differentiation. Moreover, the expressions of SREBP1 and FASN were also significantly decreased in CD38^−/−^ MEFs. Finally, the CD38 deficiency‐mediated activations of Sirt1 signalling were up‐regulated or down‐regulated by resveratrol and nicotinamide, respectively. These results suggest that CD38 deficiency impairs adipogenesis and lipogenesis through activating Sirt1/PPARγ‐FASN signalling pathway during the development of obesity.

## Introduction

Obesity is a metabolic syndrome which is associated with many diseases including type 2 diabetes, cardiovascular disease and cancer 1, 2, 3. Adipogenesis is a crucial aspect in controlling body fat mass 4. Mature adipocytes differentiated from mesenchymal stem cells (MSCs) were regulated by many transcriptional factors, like peroxisome proliferator‐activated receptor γ (PPARγ), and CCAAT/enhancer‐binding protein (CEBP)α/β 5, resulting in increases in size and number of mature adipocytes in adipose tissue. PPARγ is considered a master regulator of adipocyte differentiation. The activation of PPARγ is necessary for the expressions of adipocyte‐specific genes, such as fatty acid‐binding protein 4 (FABP4; also known as aP2) and fatty acid synthase (FASN), which facilitate the cytoplasmic storage of massive amounts of triglycerides 6. Expression of aP2 is highly induced during adipocyte differentiation and transcriptionally controlled by PPARγ agonists, fatty acids and insulin 7, 8, 9. In addition, The DNA‐binding protein CEBP interacts with the promoter of aP2 and elevates expression of the aP2 gene 10. Therefore, a better understanding the processes of regulating adipocyte development and function may provide novel targets for prevention and treatment of obesity and related metabolic diseases.

CD38 antigen is a 45‐kD type II transmembrane glycoprotein with a short N‐terminal cytoplasmic domain and along C‐terminal extracellular domain 11. It was first identified in lymphocytes as a lymphocyte‐specific antigen 12, and then, it has been found that the protein is widely expressed in a variety of cell types. The functions of CD38 are involved in numerous biological processes including egg fertilization, cell proliferation, muscle contraction, hormone secretion and immune responses. CD38 has both ADP‐ribosyl cyclase and cADPR hydrolase activities in which it cleaves NAD^+^ to generate cyclic ADP ribose (cADPR), a putative Ca^2+^ second messenger, and ADPR 13, 14, respectively. Meanwhile, CD38 can also catalyse NADP^+^ to NAADP which is also a potent trigger for Ca^2+^ mobilization. In addition, it regulates the activities of enzymes which use NAD^+^ as a substrate such as sirtuins through consuming intracellular NAD^+^ 15, 16. Numerous reports showed that CD38 was highly expressed in adipose of people with obese using gene microarray 17, 18. Barbosa *et al*. observed that CD38 deficiency protected mice from HFD‐induced obesity through increasing intracellular NAD^+^ levels and Sirt1 activity 15. However, whether CD38 affects adipogenesis and lipogenesis remains unclear.

In this study, we investigated the effects of CD38 on adipogenesis and lipogenesis using CD38^−/−^ mice and mouse embryonic fibroblasts (MEFs) derived from both CD38^−/−^ and WT mice. Our results showed that CD38 deficiency impaired adipogenesis and lipogenesis in adipose tissues.

## Materials and methods

### Chemicals and antibodies

Resveratrol, EX527, nicotinamide, cADPR, NAD^+^ and Oil Red O were purchased from Sigma‐Aldrich (St. Louis, MO). Antibodies against PPARγ, C/EBPα, SREBP1 and tubulin were purchased from Santa Cruz Biotechnology (Dallas, TX, U.S.A). Antibody against Sirt1 was purchased from Millipore (Massachusetts, U.S.A). GAPDH and FASN antibodies were purchased from Abcam (Cambridge, MA, USA). Immobolin‐P transfer membranes were obtained from Millipore (Massachusetts, U.S.A), and electrophoresis supplies were purchased from Bio‐Rad Laboratories (Hercules, California, USA).

### Mice

CD38^−/−^ mice were kindly provided by Dr. Frances E. Lund (Rochester). Animals were maintained under controlled temperature (22–24°C) and illumination (12‐hr dark/light cycle). All animals were treated in accordance with the Guide for the Care and Use of Laboratory Animals of Nanchang University, and the all experimental protocols were approved by the Ethics Committee of Nanchang University and the experiments were carried out in accordance with the approved guidelines. Eight‐week‐old male mice had free access to water and were fed either standard chow or HFDs (60% HFD, D12492; Research Diets Inc. New Brunswick, NJ,USA) *ad libitum* for 12 weeks.

### Cell culture and adipocyte differentiation

Mouse embryonic fibroblasts (MEFs) were prepared from 13.5‐day‐old embryos obtained from CD38^−/−^ or WT mice. Briefly, after dissection of head and visceral organs, embryos were minced and trypsinized for 30 min. at 37°C. Embryonic fibroblasts were then plated and maintained in DMEM with 10% foetal bovine serum, 100 U/ml penicillin and 100 mg/ml streptomycin at 37°C in an atmosphere of 5% CO2. All experiments were performed with MEFs between 15 and 20 passages. 3T3‐L1 preadipocytes were maintained in DMEM containing 10% foetal calf serum. For adipocyte differentiation, 2‐day post‐confluent cells (day 0) were treated with DMEM supplemented with 10% FBS, 0.5 mM 3‐isobutyl‐1‐methylxanthine, 1 μM dexamethasone and 1 μg/ml insulin for 2 days. Medium was changed every 2 days with DMEM containing 10% FBS and 1 μg/ml insulin until cells were used. C3H10T1/2 cells were treated with two different adipocyte differentiation protocols, adipocyte differentiation was performed by treating post‐confluent cells for 48 hrs in DMEM containing 10% foetal calf serum, 0.5 mM 3‐isobutyl‐1‐methylxanthine, 1 μM dexamethasone and 1 μg/ml insulin, and/or 0.125 mM indomethacin and/or 1 nM triiodothyronine (T3). After 48 hrs, the medium was withdrawn and replaced by medium supplemented with only insulin or both insulin and T3 until cells were used. Adipocyte differentiation of MEF is the same with 3T3‐L1, and the difference is the concentration of insulin is 10 μg/ml.

### Oil Red O staining

Lipid accumulation of differentiated adipocytes was determined by quantitative Oil Red O staining. Briefly, cells were washed twice with phosphate‐buffered saline (PBS) and fixed with 4% paraformaldehyde for 1 hr at room temperature. After washing with PBS, the cells then stained with Oil Red O for 1 hr using a 60:40 (v/v) dilution in water of a 0.5% stock solution (in isopropanol). Cells were then washed twice with distilled water, the plates were dried at room temperature, and visualized under an inverted microscope (Olympus, Tokyo, Japan). Oil red O was eluted using 100% isopropanol, and absorbance at 500 nm was measured.

### RNA isolation, cDNA synthesis and real‐time PCR

Total RNA from cells and white adipose tissue were isolated using the TRIzol method (Invitrogen, MA, USA) followed by DNase treatment. RNA was reversely transcribed using the Takara high‐capacity cDNA synthesis kit. Relative expression of mRNAs was determined after normalization with GAPDH levels using the ΔCt method. Quantitative PCR was performed using the ABI‐ViiA7 PCR machine. Primers used for real‐time PCR are shown below: PPARγ, 5‐GTGCCAGTTTCGATCCGTAGA‐3 (sense) and 5‐GGCCAGCATCGTGTAGATGA‐3 (antisense); aP2, 5‐ACACCGAGATTTCCTTCAAACTG‐3 (sense) and 5‐CCATCTAGGGTTATGATGCTCTTCA‐3 (antisense); CEBPα, 5‐TAGGTTTCTGGGCTTTGTGG‐3 (sense) and 5‐AGCCGTTAGTGAAGAGTCTCAGTTT‐3 (antisense); FASN, 5‐GGAGGTTGCTTGGAAGAG‐3 (sense) and 5‐CTGGATGTGATCGAATGCT‐3 (antisense); CD38, 5‐GAGCCTACCACGAAGCACTTTT‐3 (sense) and 5‐GGCCGGAGGATCTGAGTGTA‐3 (antisense); GAPDH, 5‐AGCCAAAAGGGTCATCATCT‐3 (sense) and 5‐GGGGCCATCCACAGTCTTCT‐3 (antisense).

### Western blotting

For Western blot analysis, cells or tissues were lysed in RIPA buffer (0.5% NP‐40, 0.1% sodium deoxycholate, 150 mM NaCl, 50 mM Tris‐Cl, pH 7.5). Lysates were centrifuged at 13000 *g* for 10 min. at 4°C and protein concentration determined using the Bradford reagent (Bio‐Rad) with BSA as standard. Lysates were resolved by SDS‐PAGE, transferred to PVDF membrane (Millipore) and probed with indicated antibodies.

### Statistical analysis

For tissue study, data are representative of at least three independent mice with a similar result. The Western blot images were semi‐quantified with the Image Lab 3.0 program. Statistical analysis of the data was performed using Student's *t*‐test. All data are presented as mean ± S.E.M. Statistical significance was set at *P < 0.05, **P < 0.01.

## Results

### CD38 deficiency impairs adipogenesis *in vivo*

**Figure 1:**
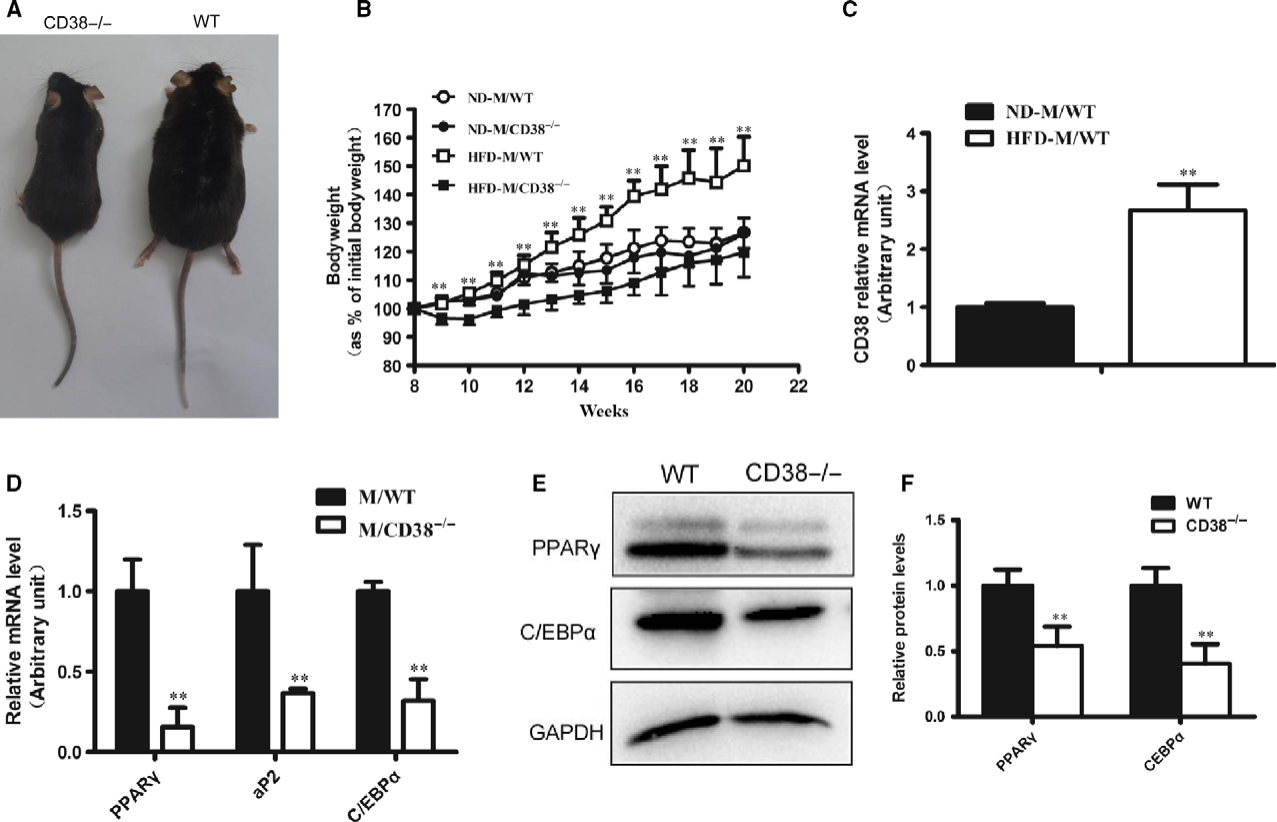
Effects of CD38 deficiency on adipogenesis *in vivo*. (**A**) An image of the mice fed with high‐fat diet (HFD). Eight‐week‐old wild‐type (WT) and CD38 knockout (CD38^−/−^) mice were fed with normal diet (ND) or HFD for 12 weeks. (**B**) The gain curves of bodyweight of mice. The bodyweights of the WT and CD38^−/−^ mice which were fed with ND or HFD were measured every week, and the percentage of the bodyweight gain was represented with the initial bodyweight of the mice as a control. (**C**) The mRNA expressions of CD38 in WT mice. The total RNAs were extracted from white adipose tissues of WT mice that were fed with ND or HFD for 12 weeks, and the mRNA expressions of CD38 were assessed by q‐PCR. (**D**) Quantitative analysis of the expressions of the adipogenic genes. The mRNA expressions of PPARγ, aP2 and C/EBPα were analysed by q‐PCR with the adipose tissues of WT and CD38^−/−^ mice after feeding HFD of 12 weeks. (**E**) Expressions of PPARγ and C/EBPα proteins in adipose tissues. The proteins were determined by Western blot analysis with white adipose tissues of WT and CD38^−/−^ mice which were fed with HFD. (**F**) The protein levels of PPARγ and C/EBPα were quantified by Western blot analysis from three different experiments. The values are expressed as the mean ± S.E. *n* = 5–6. *P < 0.05, **P < 0.01.

The bodyweights of CD38^−/−^ mice were significantly lighter than those of WT mice when the both mice were fed with HFD for 12 weeks (Fig. 1A and B). To determine whether CD38 expression might be altered in obesity, we then assayed CD38 expression in white adipose tissue from WT mice fed with HFD. The results showed that CD38 expression was markedly increased in adipose tissue from the mice fed with HFD compared with the mice fed with normal diet (Fig. 1C). Meanwhile, the mRNA and protein expressions of adipogenic genes including PPARγ, aP2 and CEBPα were significantly decreased in adipose tissue from CD38^−/−^ mice fed with HFD (Fig. 1D–F). Collectively, these data provide evidence that CD38 deficiency impairs adipocyte differentiation in mice fed with HFD.

### CD38 deficiency impairs adipogenesis *in vitro*

**Figure 2:**
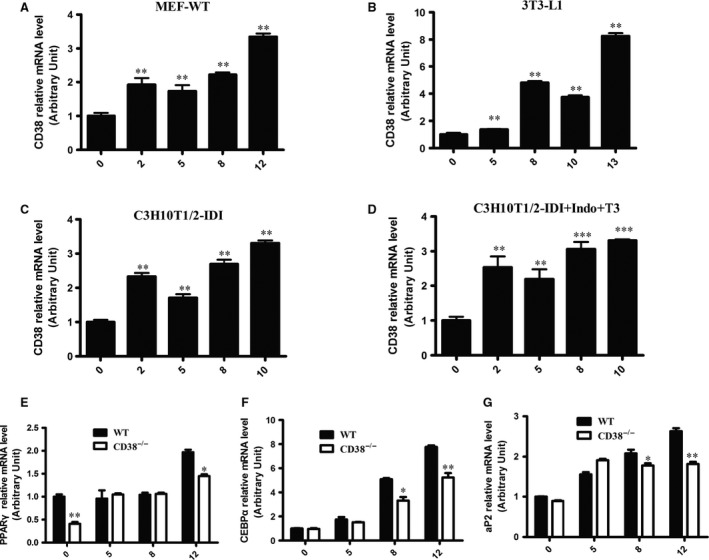
Effects of CD38 deficiency on adipogenesis *in vitro*. Primary mouse embryonic fibroblasts (MEFs) were delivered from CD38^−/−^ and WT mice, and the MEFs were differentiated into adipocytes induced by IBMX, dexamethasone and insulin for 48 hrs followed by treatment of insulin alone every 2 days. (**A**–**D**) The mRNA expressions of CD38 in cells. The mRNA expressions were measured by qPCR using MEFs from WT mice, 3T3‐L1 and C3H10T1/2 cells (**A**–**C**) during induction of adipocyte differentiation with the standard induction cocktail, respectively, or in C3H10T1/2 cells with a brown adipogenic induction cocktail (**D**), the mRNA expressions are expressed relative to Day 0 set at 100%. (**E**–**G**) The mRNA expressions of adipogenic genes. The mRNA levels of adipogenic genes including PPARγ (**E**), CEBPα (**F**) and aP2 (**G**) were evaluated by q‐PCR after stimulation of the adipose differentiation induction with WT and CD38^−/−^ MEFs. The values are expressed as the mean ± S.E. from three independent experiments. *n* = 3. *P < 0.05, **P < 0.01 and ***P < 0.001.

**Figure 3:**
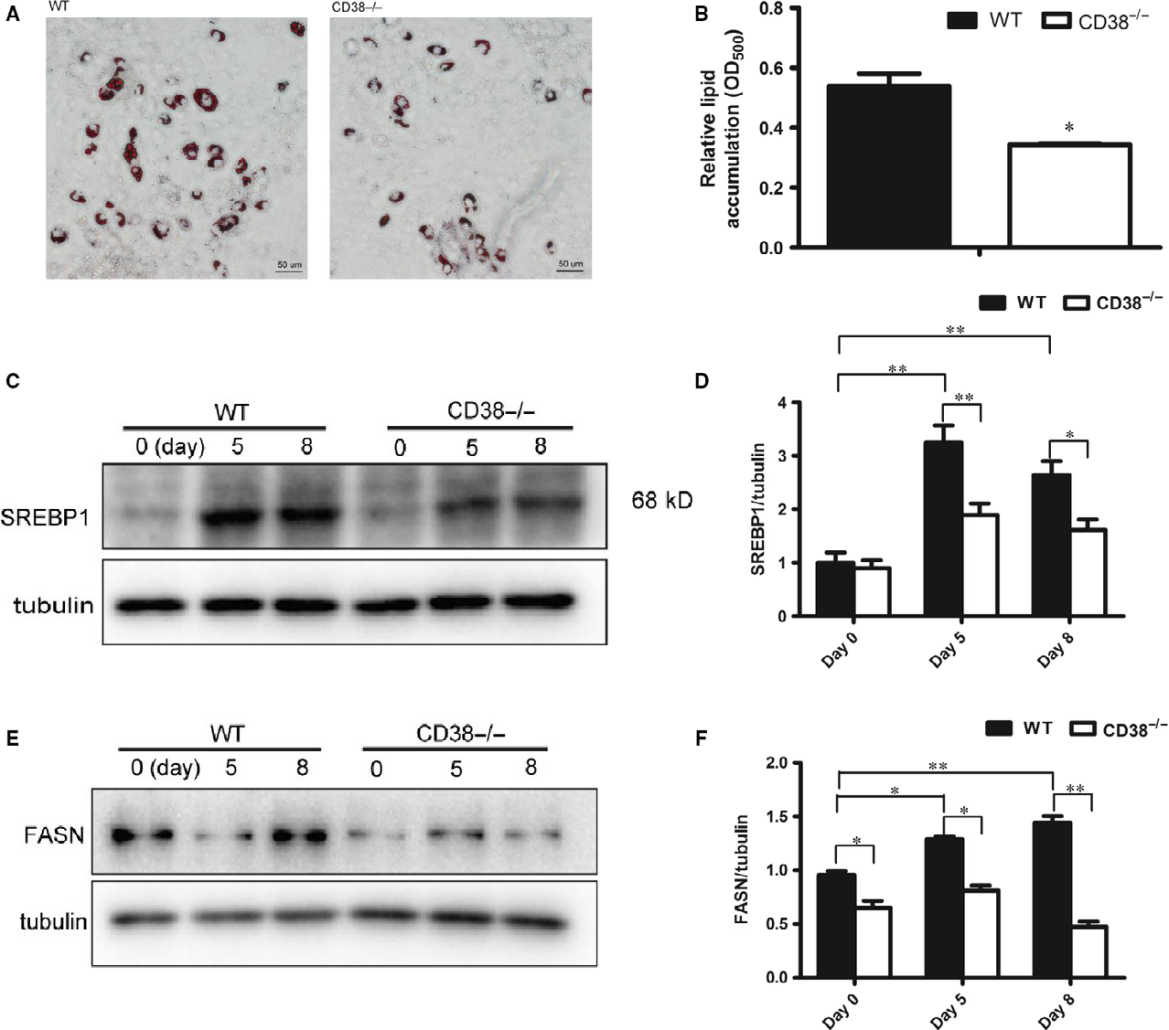
Effects of CD38 deficiency on Lipogenesis in MEFs. (**A**) Images of lipid in adipose cells. Lipid accumulations were evaluated by Oil Red O staining at 12 days of adipocyte differentiation induction. (**B**) Quantitative analysis of lipid formation. The relative lipid accumulation was quantitatively analysed by measuring absorbance at 500 nm. (**C, D**) Protein expression of SREBP1. The total cellular extracts were prepared from WT and CD38^−/−^ MEFs after adipocyte differentiation induction, and the levels of proteins were analysed by Western blotting with antibodies against SREBP1 and tubulin was used as an internal control. Semi‐quantitative analysis of SREBP1 (**D**) was performed by calculating the density of Western blot bands. (**E, F**) Protein expression of FASN. Semi‐quantitative analysis of FASN (**F**) was performed by calculating the density of Western blot bands. (200×. scale bar 50 μm). The values are expressed as the mean ± S.E. from three independent experiments. *n* = 3. *P < 0.05, **P < 0.01.

To clarify whether CD38 affects adipogenesis *in vitro*, we first examined the expressions of CD38 in several cell lines when adipocyte differentiation was induced. Our results showed that CD38 expressions were dramatically up‐regulated during adipogenesis in MEFs (Fig. 2A) and 3T3‐L1 (Fig. 2B) in a time‐dependent manner. We also examined the expressions of CD38 during adipocyte differentiation using C3H10T1/2 cells with two different protocols: insulin + dexamethasone + isobutylmethylxanthine (IDI, the standard induction cocktail) and IDI + indomethacin (IDI + Indo + T3, a brown adipogenic induction cocktail). The results showed that CD38 expressions were significantly increased after 2 days of differentiation induction and reached to the highest at day 10 in both protocols (Fig. 2C and D). To further investigate the effects of CD38 on adipogenesis, we detected the alterations of the adipogenic genes during differentiation using WT and CD38^−/−^ MEFs. The results showed that although the mRNA expressions of PPARγ, CEBPα and aP2 in both MEFs from WT and CD38^−/−^ mice were increased during adipocyte differentiation, the expressions of these genes in CD38^−/−^ MEFs were remarkably lower than that of WT MEFs (Fig. 2E–G). In addition, we also found that PPARγ mRNA expression in CD38^−/−^ MEFs was much lower than WT MEFs before differentiation induction (Fig. 2E). Taken together, these results suggested that CD38 was essential in adipogenesis *in vitro*.

### CD38 deficiency impairs lipogenesis in MEFs

To explore whether anti‐obesity of CD38 deficiency was also involved in lipogenesis, the alternations of lipid accumulation and the expressions of the related genes were also examined with MEFs from WT and CD38^−/−^ mice. The results showed that the lipid accumulation which was evaluated by Oil Red O staining and measurement of absorbance at 500 nm with MEFs derived from CD38^−/−^ mice were markedly inhibited after 12 days of insulin stimulation compared with that of MEFs from WT mice (Fig. 3A and B). As SREBP1 is a transcription factor that controls fatty acid synthase (FASN), and functions as an additional regulator of adipogenesis in parallel with C/EBPα and PPARγ pathways, the expressions of SREBP1 and FANS were detected in WT and CD38^−/−^ MEFs. Our results showed that although the expressions of SREBP1 in WT and CD38^−/−^ MEFs were significantly elevated when differentiation induction, the elevations of SREBP1 in CD38^−/−^ MEFs were lower than that in WT MEFs (Fig. 3C and D). Furthermore, we also observed that the expressions of SREBP1‐mediated FASN were down‐regulated in CD38^−/−^ MEFs compared with WT MEFs during adipocyte differentiation induction (Fig. 3E and F). These results indicated that CD38 deficiency also impaired lipogenesis in adipose tissues.

### CD38 deficiency activates NAD^+^/Sirt1 pathway

**Figure 4:**
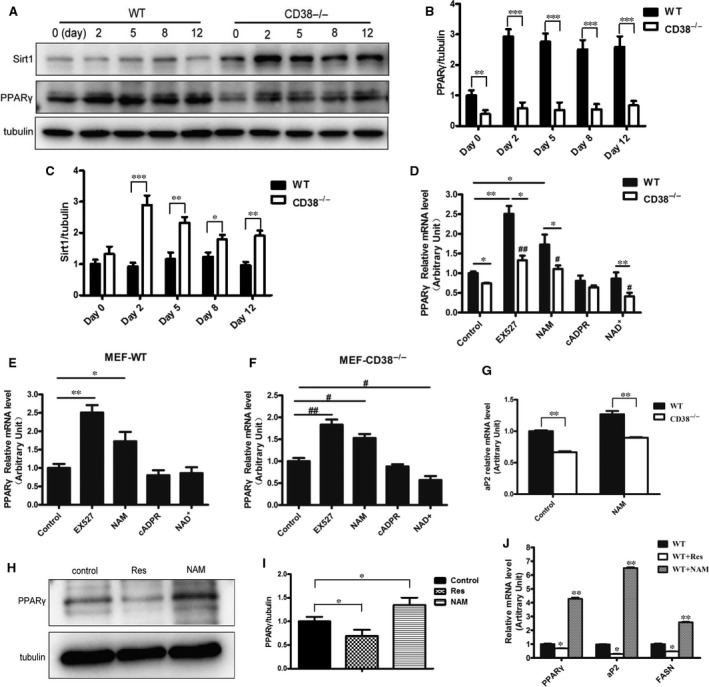
Effects of Sirt1 on CD38 deficiency‐mediated inhibition of adipocyte differentiation. (**A**) The protein expressions of Sirt1 and PPARγ. The protein levels of Sirt1 and PPARγ were significantly up‐regulated or down‐regulated, respectively, after stimulation of the adipose differentiation process in CD38^−/−^ MEFs and tubulin was loaded as an internal control. (**B**–**C**) Semi‐quantitative analysis of PPARγ and Sirt1 was performed by calculating the density of Western blot bands. (**D**–**F**) The mRNA expressions of PPARγ from wild‐type and CD38^−/−^ MEFs treated with various reagents. The mRNA levels of PPARγ were determined by qRT‐PCR from wild‐type and CD38^−/−^ MEFs with EX527 (a Sirt1‐specific inhibitor, 25 μM), Nicotinamide (a Sirt1 non‐specific inhibitor, 10 mM), cADPR (a CD38‐derived metabolite, 0.5 μM) and NAD ^+^ (a CD38 substrate, 1.0 mM) for 24 hrs after induction of adipocyte differentiation (**D**). The relative mRNA expressions of PPARγ in wild‐type (**E**) or CD38^−/−^ (**F**) MEFs were compared with control groups when the cells were treated with various stimuli. (**G**) The mRNA expressions of aP2 in MEFs. The mRNA expressions of aP2 were determined by qRT‐PCR after treatments with nicotinamide, a Sirt1 inhibitor, in WT and CD38^−/−^ MEFs. (**H**–**I**) The protein expressions of PPARγ. The protein levels of PPARγ were determined by Western blot analysis with or without resveratrol (50 μM) or nicotinamide (10 mM) in 3T3‐L1 adipocytes, and tubulin was loaded as an internal control. Semi‐quantitative analysis of PPARγ (**I**) was performed by calculating the density of Western blot bands. (**J**) The mRNA expressions of PPARγ, aP2 and FASN. The mRNA levels of PPARγ, aP2 and FASN from 3T3‐L1 adipocytes were evaluated by RT‐PCR at day 12 after the initiation of adipogenesis. The values are expressed as the mean ± S.E. from three independent experiments. *n* = 3. *P < 0.05, **P < 0.01 and ***P < 0.001. vs WT group. ^#^ *P* < 0.05, ^##^ *P* < 0.01 vs CD38^−/−^ group. [Correction added on 11 September 2017, after first online publication: An incorrect version of Figure 4 was published previously and has been corrected in this version.]

**Figure 5:**
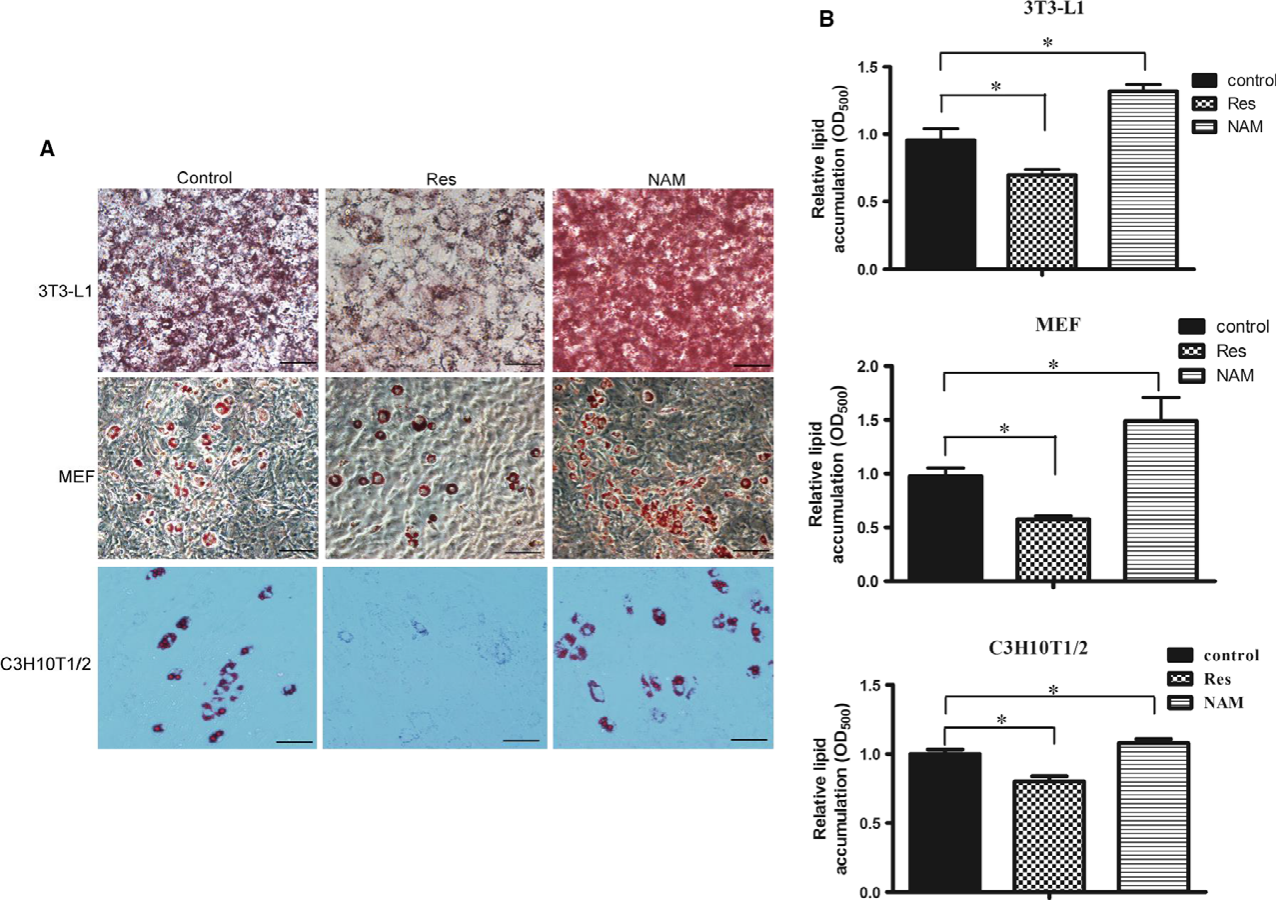
Effects of Sirt1 on CD38 deficiency‐mediated inhibition of lipogenesis. (**A**) Images of lipid formation treated with resveratrol and nicotinamide. Lipid formations were assessed by Oil Red O staining and visualized by bright field light microscopy in 3T3‐L1, MEFs and C3H10T1/2 cells after treated with resveratrol (a Sirt1 activator) and nicotinamide (a Sirt1 inhibitor) at day 12 during induction of adipocyte differentiation. (**B**) Quantitative analysis of lipid formation. The relative lipid accumulations were quantitatively assessed by measuring absorbance at 500 nm. (200×. scale bar 50 μm). The values are expressed as the mean ± S.E. from three independent experiments. *n* = 3. *P < 0.05.

It has been reported that the NAD^+^ levels and Sirt1 activities were significantly increased in a variety of tissues/cells of CD38^−/−^ mice 16. To explore the molecular mechanisms underlying CD38 deficiency‐mediated inhibition of obesity, we detected the protein expression levels of Sirt1 and the target gene PPARγ which was known to promote adipocyte differentiation. Western blot analysis showed that PPARγ, a master adipogenic regulator, was significantly decreased in CD38^−/−^ MEFs during differentiation (Fig. 4A and B). However, we observed robust activation of the Sirt1 in CD38^−/−^ MEFs compared to WT especially during the first 2 hrs of adipocyte differentiation induction (Fig. 4A and C). Furthermore, to further clarify the involvement of the Sirt1 signalling pathway in the anti‐adipogenic effects of CD38 deficiency, the MEFs from WT and CD38^−/−^ mice were treated with EX527 (a Sirt1 specific inhibitor, 25 μM), nicotinamide (a Sirt1 non‐specific inhibitor, 10 mM), cADPR (a CD38‐derived metabolite, 0.5 μM) and NAD^+^ (a CD38 substrate, 1.0 mM) during adipocyte differentiation induction. The results showed that EX527 and NAM markedly increased PPARγ expressions in both wild‐type and CD38^−/−^ MEFs, whereas the expressions of PPARγ with treatment of EX527 were much higher than those of treating with NAM in both MEFs, suggesting that the PPARγ‐mediated adipogenesis is mainly inhibited by Sirt1 (Fig. 4D). In addition, NAD^+^ significantly reduced PPARγ expression in CD38^−/−^ MEF cells, but not in wild‐type MEF cells, indicating that the NAD‐mediated Sirt1 activation is also involved in the adipogenesis (Fig. 4D). However, the CD38‐derived metabolite cADPR had no effects on the expressions of PPARγ in both wild‐type and CD38^−/−^ MEF cells, demonstrating that CD38‐mediated cADPR‐Ca^2+^ signalling pathway might not be an important factor for adipogenesis during adipocyte differentiation induction (Fig. 4D). Interestingly, although the absolute expressions of PPARγ in wild‐type MEF cells with treatment of EX527 and NAM were much higher than those in CD38^−/−^ MEF cells the increased ranges were comparable in both MEF cells (Fig. 4E and F). In addition, the results also showed that nicotinamide increased the mRNA expressions of aP2 in both cell types compared to control, whereas the expressions of aP2 in CD38^−/−^ MEFs were markedly reduced than that of WT MEFs (Fig. 4G). Consistent with the results, there were the similar alternations of the expressions of PPARγ, aP2, and FASN when treated with nicontinamide in 3T3‐L1 cells, whereas resveratrol (a Sirt1 activator) inhibited the expressions of these genes (Fig. 4H–J). In addition, we also examined the lipogenic effects of resveratrol and nicotinamide on three cell lines, respectively. Oil Red O staining showed that resveratrol resulted in decrease in lipid accumulation compared to the control, but nicotinamide increased lipogenesis (Fig. 5A) and the accumulations were quantitatively summarized in Figure 5B. Taken together, our data further demonstrated that anti‐obesity of CD38 deficiency was involved in inhibitions of adipogenesis and lipogenesis probably by activating Sirt1‐mediated PPARγ inhibition.

## Discussion

Development of obesity arises from increased size of individual adipose cells caused by lipid accumulation, and/or from increased number of adipocytes upon differentiation of preadipocytes into mature adipocytes under the appropriate hormonal stimuli 19. It has been reported that deletion of CD38 gene protected mice from HFD‐induced obesity 15. In the present study, we also confirmed that CD38 deficient mice were significantly resistant to HFD‐induced obesity. Furthermore, we found that the expressions of CD38 were markedly elevated in WT mice fed with HFD. These results suggested that CD38 played a critical role in HFD‐induced obesity in mice. However, whether the protection of CD38 deficient mice on the obesity induced by HFD is involved in inhibitions of adipogenesis or/and lipogenesis is still unknown. To answer this question, we first examined the expressions of the adipogenic genes including PPARγ, FABP4 (aP2) and C/EBPα in adipose tissues isolated from CD38^−/−^ and WT mice fed with HFD. Our results showed that the expressions of all these genes were significantly attenuated in CD38^−/−^ mice fed with HFD compared with those of WT mice, suggesting that the protection of CD38 deficiency on obesity may be associated with inhibitions of adipocyte differentiation through repressing the expressions of PPARγ and C/EBPα in adipose tissues.

To further explore the mechanisms of CD38 in adipocyte differentiation, we first carried out the experiments of induction of adipocyte differentiation *in vitro* with MEFs, 3T3‐L1 and C3H10T1/2, the mesenchymal pluripotent cells. Our results showed that the expressions of CD38 were significantly increased in MEFs derived from WT mice, 3T3‐L1 and C3H10T1/2 during adipocyte differentiation induction in a time‐dependent manner. Importantly, although the expressions of PPARγ, aP2 and C/EBPα in both MEFs from CD38‐deficient and WT mice were increased during induction of adipocyte differentiation, the elevations of the expressions of the genes in CD38‐deficient MEFs were significantly less than that of WT MEFs. In addition, we also observed that accumulations of lipid were markedly decreased in MEFs from CD38 null mice compared with that of WT mice. All of these results demonstrated that alternations of CD38 expression were associated with adipogenesis and lipogenesis *in vivo* and *in vitro*. It has been reported that C/EBPβ and C/EBPδ expressions were induced in the early of the differentiation in 3T3‐L1 preadipocytes 20, whereas the expressions of PPARγ and C/EBPα were induced in the terminal differentiation 21. It has been identified that PPARγ is a master regulator of adipocyte differentiation and metabolism 22, in which the expression of PPARγ was promoted by C/EBPβ and C/EBPδ through binding to two C/EBP binding sites in the promoter of PPARγ2 23, 24. Therefore, C/EBPβ and C/EBPδ appear to play an important role in the initiation of the adipogenic cascade 20, whereas PPARγ and C/EBPα may play the most prominent roles in the terminal differentiation 21. Except as the key transcription factor of the adipocyte differentiation, PPARγ is also an important target of diabetes drugs. It has been reported that CD38 plays an essential role in intracellular Ca^2+^ mobilization by cADPR for insulin secretion 25. Furthermore, researchers demonstrated PPARγ‐mediated insulin sensitization by enhancing NAADP production through upregulation of CD38 in adipocytes, and CD38^−/−^ completely blocked the effects 26. Besides, fatty acid‐binding protein 4 (FABP4 or aP2) which is mainly expressed in adipocytes and macrophages also plays an important role in lipolysis for transportation of fatty acids and in the development of insulin resistance and atherosclerosis in relation to meta inflammation 27. Moreover, aP2 is one the most abundant proteins ever found in mature adipocytes which accounts for up to 6% of total cytosolic proteins in cultured differentiated fat cells 28. It has been reported that aP2 expression is controlled by PPARγ 29. In contrast, aP2 may regulate the transcriptional activation of PPARγ by promoting PPARγ continuous shuttling between nucleus and cytoplasm 30. In some studies in cell culture, aP2 can transfer to the nucleus and interact with, and potentially deliver ligands to PPARγ 29, 30, 31, but the functional consequences of this interaction are unknown. Except as a small intracellular protein, in fact, emerging data have strongly linked serum aP2 that is preferentially produced and released from adipocytes with metabolic disease risk in humans 32, 33. In the present study, we observed that CD38 deficiency significantly reduced the expressions of PPARγ, C/EBPα and aP2 in the late of the differentiation induction, which were consistent to the results that PPARγ and C/EBPα were transcription factors regulating late adipogenesis 21. Thus, our results demonstrated that deletion of CD38 gene inhibited adipocyte differentiation through mainly influencing the terminal stage of differentiation.

Recent studies indicated that SREBP1c is one of the earliest transcription factors in adipocyte differentiation and is required to induce expressions of C/EBPβ and C/EBPδ 34. Moreover, SREBP1c appears to contribute to the expression of PPARγ and the production of endogenous PPARγ ligands during adipocyte differentiation 35, 36. SREBP1c can also directly regulate the expression of several key genes of lipid metabolism, such as fatty acid synthase (FASN) and acetyl‐CoA carboxylase (ACC), the two rate‐limiting enzymes controlling *de novo* lipogenesis. The expression of FASN gene was also governed by PPARγ and involved in the facilitated conversion of FFA into TG in adipocytes, thus contributing to adipocyte hypertrophy and visceral adiposity in HFD‐fed obesity mice 37. In our study, the expression and activity of SREBP1 and its target gene FASN were significantly attenuated in CD38^−/−^ MEFs, indicating that CD38 deficiency not only inhibits adipocyte differentiation but also suppresses lipogenesis.

Sirt1, a NAD^+^‐dependant protein deacetylase, is increasingly referred to as a master metabolic regulator due to its ability to modify and control numerous transcription factors involved in the whole‐body metabolic homoeostasis. In mature fat cells, Sirt1 has been shown to repress PPARγ, by docking to the negative cofactors of the nuclear receptor NcoR, resulting in mobilization of fat in fully differentiated adipocytes and a decrease in adipocyte formation from preadipocytes 38. In addition, Jin Q *et al*. found that C/EBPα promoted Sirt1 expression by directly binding to the promoter of Sirt1 39. It has been reported that there was a significant elevation of intracellular/tissue NAD^+^ level along with an increase of Sirt1 activity in a variety of cell types or tissues of CD38^−/−^ mice compared with WT mice 16. CD38 is a main cellular NADase in mammalian tissues and the activities of many enzymes including sirtuins which use NAD^+^ as a substrate in the most tissues are markedly elevated from CD38^−/−^ mice. In the present study, we observed that the expressions of Sirt1 in CD38‐deficient MEFs were significantly increased during induction of adipocyte differentiation and the expressions of PPARγ, aP2 and FASN mRNA were remarkably down‐regulated by resveratrol (an activator of sirt1) and up‐regulated by nicotinamide (an inhibitor of sirt1) in CD38^−/−^ MEFs and 3T3‐L1 cells, respectively, indicating that CD38 deficiency suppressed adipogenesis and lipogenesis through activating Sirt1 signalling pathway. More recently, Ponugoti *et al*. found that Sirt1 directly inhibited SREBP1c activity through deacetylating lys‐289 and lys‐308 of SREBP1c, resulting in decreases of its stability and its association with its lipogenic target gene promoters 40. More importantly, our results showed that although EX527 (a Sirt1 specific inhibitor) and NAM markedly increased PPARγ expressions in both wild‐type and CD38^−/−^ MEFs, the expressions of PPARγ with treatment of EX527 were much higher than that of treating with NAM in both MEFs, suggesting that the PPARγ‐mediated adipogenesis is mainly inhibited by Sirt1. In addition, we also found that the CD38‐derived metabolite cADPR had no effects on the expressions of PPARγ in both wild‐type and CD38^−/−^ MEFs cells, indicating that cADPR might not be an important factor for adipogenesis during adipocyte differentiation induction.

Collectively, our data indicated that CD38 may act as a key factor to participate in adipogenesis and lipogenesis of adipose tissues through regulating Sirt1‐mediated signalling pathway. On the one hand, CD38 deficiency significantly promotes Sirt1 activity by elevating the levels of intracellular NAD^+^. On the other hand, the increase in CEBPα activity during adipocyte differentiation induction enhances the expressions of Sirt1, resulting in Sirt1‐mediated inhibition of PPARγ and SREBP1c in adipogenesis and lipogenesis, respectively. Therefore, based on the results of this study, we concluded that CD38 deficiency‐mediated activation of Sirt1 signalling pathway plays a critical role in the development of obesity.

## Conflict of interest

The authors declare no competing financial interests.
